# Performance Analysis of Emergency Room Episodes Through Process Mining †

**DOI:** 10.3390/ijerph16071274

**Published:** 2019-04-10

**Authors:** Eric Rojas, Andres Cifuentes, Andrea Burattin, Jorge Munoz-Gama, Marcos Sepúlveda, Daniel Capurro

**Affiliations:** 1 Department of Computer Science, School of Engineering, Pontificia Universidad Católica de Chile, Santiago 7820436, Chile; alcifuen@uc.cl (A.C.); jmun@uc.cl (J.M.-G.); marcos@ing.puc.cl (M.S.); 2 Department of Internal Medicine, School of Medicine, Pontificia Universidad Católica de Chile, Santiago 8331150, Chile; dcapurro@med.puc.cl; 3 Software and Process Engineering, Technical University of Denmark, 2800 Kgs. Lyngby, Denmark; andbur@dtu.dk

**Keywords:** Process Mining, healthcare, Emergency Room

## Abstract

The performance analysis of Emergency Room episodes is aimed at providing decision makers with knowledge that allows them to decrease waiting times, reduce patient congestion, and improve the quality of care provided. In this case study, Process Mining is used to determine which activities, sub-processes, interactions, and characteristics of episodes explain why some episodes have a longer duration. The employed method and the results obtained are described in detail to serve as a guide for future performance analysis in this domain. It was discovered that the main cause of the increment in the episode duration is the occurrence of a loop between the Examination and Treatment sub-processes. It was also found out that as the episode severity increases, the number of repetitions of the Examination–Treatment loop increases as well. Moreover, the episodes in which this loop is more common are those that lead to Hospitalization as discharge destination. These findings might help to reduce the occurrence of this loop, in turn lowering the episode duration and, consequently, providing faster attention to more patients.

## 1. Introduction

Performance measurements of Emergency Room (ER) episodes are important, mainly because of the information that can be provided to identify the behavior of episodes with extended waiting times for patients awaiting ER care. Identifying opportunities for improvement in ER episodes will help reducing waiting times, directly improving the quality of services provided and reducing overcrowding for future patients [1,2].

The American College of Emergency Physicians defines Emergency medicine as the medical specialty in charge of diagnosis and treatment of unforeseen illness or injury in an Emergency Room [3]. An episode of attention in the ER will include the initial evaluation of the patient, the diagnosis and treatment, and, finally, the required actions to provide a positive outcome [4].

Our approach to study ER episode performance is based on Process Mining as the main component to identify, discover, and analyze activities executed during ER episodes. Process Mining is a research discipline that aims to extract knowledge about processes based on data stored in the databases of corporate information systems, in this case Hospital Information Systems (HIS) [5,6,7,8]. Process execution data is extracted as event logs, which contain all activities executed for all particular process instances, i.e., for all episodes. Process mining tools and techniques allow to discover process models, verify conformance, discover organizational patterns, and check process performance in any hospital [5]. In this context, Process Mining is proposed as a complement to other methodologies that are used to analyze process performance from a data-oriented perspective rather than a process-oriented one. ER episodes have been analyzed using simulation, data mining along with process mining [9,10], among others.

Data mining is used to find patterns and understand the causes of certain process behaviors while, on the other hand, process mining helps to understand how processes are currently being performed.

On the other hand, process simulation has a process-oriented approach and it is a powerful method to estimate the eventual effects of changes in the process design on the process performance. However, it does not allow to understand specific issues that might be affecting the current process performance, since it does not allow to delve into detailed historical data.

In this article we describe a case study in which Process Mining has been used to analyze the performance of ER episodes, so as determine which activities, sub-processes, interactions, and characteristics of episodes explain why some episodes have a longer duration.

Previous research has been conducted to study Emergency Rooms, where process mining has been applied to analyze process execution [10,11,12,13,14]. The studies have given insights in the care process and the flow of activities during ER episodes (for example, medication administration or discharge activities). The first case is a study in a hospital in Portugal where a software suite was defined to extract data, build an event log, and discover processes in a medical center [10]. A specific case study was done using ER data, but the solution is a general one. It includes clustering techniques and Markov Model Chains. The second study is exploratory and was conducted in four Australian hospitals [11], where data were extracted, an event log was built, and discovery techniques of process mining were applied. This study was extended and complemented to include further analysis to understand the differences between the hospitals and their process executions from the performance point of view [15]. Their analysis focuses on the differences between the same process on four hospital settings, but it does not study internal differences in the process executed at each hospital. The third one proposes a methodology based on frequently-posed questions, and provides a case study, but did not include a performance analysis [12].This methodology is the basis of the applied method in the presented case study. The fourth one proposes an interaction model as part of an organizational analysis of the emergency room process, but did not include a performance analysis either [13]. In the fifth study, the authors provide an introduction to process mining in the ER, providing a case study that includes the application of discovery techniques and an exploratory analysis of performance using Petri nets [14]. From the performance point of view, the study only provided a short example of the application of a performance technique on a Petri net.

In the past, other researchers have conducted Emergency Room performance analysis [16,17], but those studies did not include the use of process mining techniques to conduct performance analyses of ER episodes.

Previous research has analyzed ER performance from the Operations Research (OR) point of view. Waiting times, the efficient use of resources, the quality of planning and scheduling procedures have been studied; a detailed overview is provided in [18]. OR tends to focus on the analysis of mathematical models ranging from linear programming and project planning to queueing models, Markov chains, and simulation [5].

The proposed focus of our study is process-oriented and data-centric. Process-oriented because it describes the episodes as a sequence of activities (and sub-processes) that are executed in a specific order to provide care to a patient. Data-centric because it is based on real data from the ER episodes executed during a specific timeframe. Using real data, the details of the executed process can be studied, so as to identify activities, sub-processes, or relationships among them, that explain the slow execution of some ER episodes.

This article is an extended version of our article [19]. In this version, we provide a more detailed description of the case study and further results are presented: more details were added illustrating how the duration of the episodes varies according to the severity determined by the patient’s original severity classification (Triage); an analysis by discharge disposition was included; for the discharge disposition that presented a greater number of loops (hospitalization), the main diagnoses in which loops occur were analyzed.

The structure of the article is the following: Section 2 defines the research objectives of this study. Section 3 describes the proposed method. Section 4 describes a case study. Section 5 provides the results of the case study and Section 6 includes the discussion. Finally, conclusions an future work are highlighted in Section 7.

## 2. Objectives

Through this study we seek to reach the following objectives. First, analyzing ER episodes behavior using a comprehensively described process mining method. Second, applying this method to a case study, determining which activities, sub-processes, and their interactions in the ER episode are linked to it getting stalled and having longer episode duration. Third, identifying any existing relationships between characteristics of the episode activities or sub-processes, and the fact the episode is taking longer than expected. By achieving these goals, we provide hidden information to decision makers that will allow them to take action over existing process inefficiencies, so as to design interventions that might decrease waiting times, reduce patient congestion, and increment the quality of care.

In order to perform these diagnoses, our work proposes a method to analyze the process at different levels. A low level analysis includes every sequence of executed activities towards the identification of sub-processes, and a high level analysis includes sub-processes and their relationships. Without this approach, the process remains difficult to analyze due to its flexible and complex nature.

## 3. Methods

In the past, several methodologies have been proposed that have been used to generate process mining analysis in healthcare. Some of the most used are: L* Cycle [5], PM2 [20], Clearpath Method [21], Question driven methodology [12], among others. All of them provide steps and guidelines on how to properly execute the process mining analysis.

For the process performance analysis of ER episodes, a 6-phase method is proposed and described in this section. This method is based on the methodology proposed in [12], which provides a detailed strategy to use process mining in healthcare. That methodology provides a step by step guide on how to apply process mining in healthcare. The method presented in this paper additionally provides the capability of aggregating activities so as to improve the analysis by sub-process.

**Figure 1:**
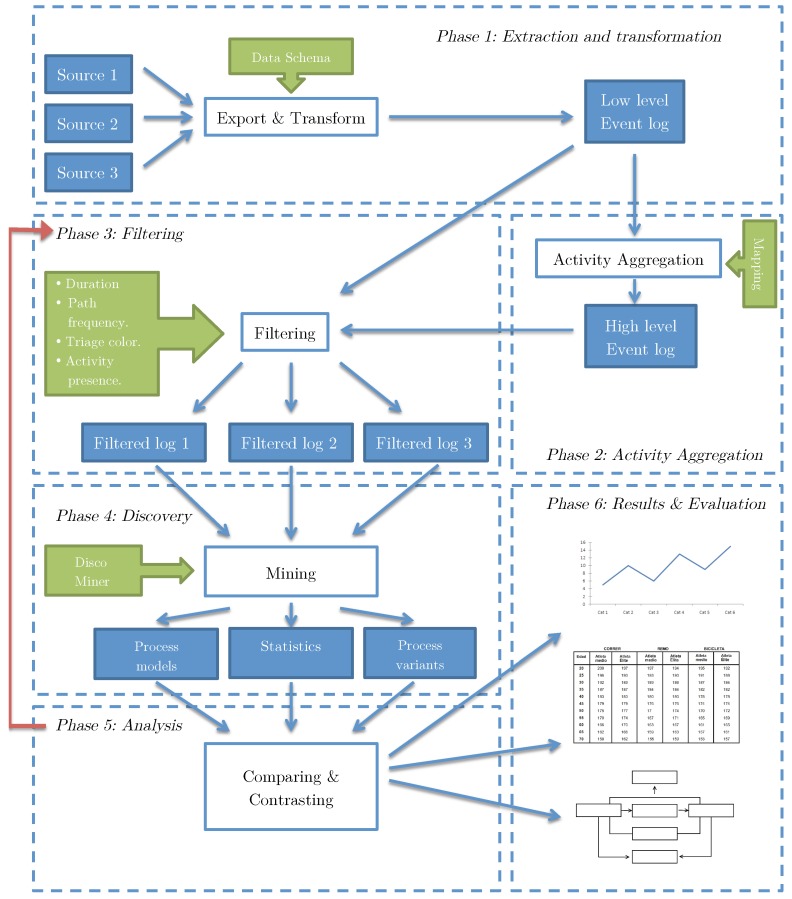
Proposed 6-phase Method. The arrow from Phase 5: Analysis to Phase 3: Filtering, represents the possibility to go back to filter the event log again to generate more specific process models.

The phases include Extraction (obtaining and processing raw data to create a low level event log), Activity Aggregation (building high level event logs based on the previously generated low level event log), Filtering (applying tools to filter the event log by different attributes), Discovery (discover a process model from the filtered event log and getting information about process variants and statistics from the discovered model), Analysis (comparing, examining and explaining discovered models), and Results and Evaluation. Figure 1 shows the dependencies between these 6 phases, which will be described in the following subsections.

### 3.1. Phase 1: Extraction and Transformation

Emergency Room episodes are supported by clinical and non-clinical activities that are executed by different types of resources (physicians, nurses, technical specialists, administrative staff). Each of these activities corresponds to an episode, and are registered in Hospital Information Systems (HIS) [6,22].

HIS records are used at ER to assemble a patient’s clinical history and enable the communication between different staff members to expedite emergency care. These records are used to build an ER event log to then discover and analyze process models. Obtaining the data to analyze may not be simple depending on the data access restrictions of the country of origin (e.g., Chilean law N°19,628, 1999).

HIS store these records as events (or activities) that include all the necessary data to create an event log to perform process mining analysis. An event log is a file record that provides an audit trail that can be used to understand the system activities. In this phase, HIS records are extracted from different sources (databases, repositories, etc.) and transformed to a standard event log format that is readable by process mining tools like CSV, XLS, XES [23] (XES was approved as the IEEE standard in 2017), among others. To perform this transformation, Disco (Fluxicon) (http://www.fluxicon.com/disco) is a fast and reliable tool that transforms historical records to a low level event log. To provide enough information to perform the analysis, it is necessary to incorporate specific data to the event log besides the minimum data required to build the process model, which are Activity Name, Timestamp, and Episode ID. An event log schema designed for this particular case study is provided in Table 1.

### 3.2. Phase 2: Activity Aggregation

**Figure 2:**
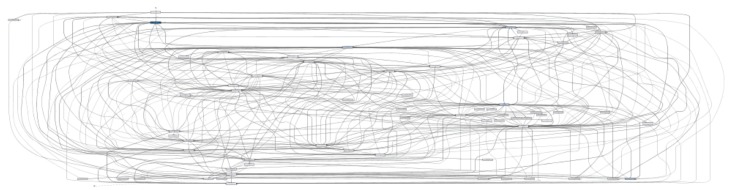
Example of spaghetti process.

The complexity of the ER episodes is defined by the amount of different activities involved in one process instance and the lack of process structure: a classic example of a spaghetti process [5]. Spaghetti processes are unstructured models with several activities connected to each other and are difficult to visualize because of the amount of information that is shown when represented as a graphical model. An example is presented in Figure 2. Therefore, it becomes necessary to take the event log to a higher level of abstraction that offers a more general view of the process in terms of the process flow and structure. Having the event log at a lower level will provide more detailed information about the activities but no sub-process analysis can be executed because of its spaghetti nature. The result is an event log that contains sub-processes rather than activities as its basic unit, which makes inspection and analysis more accessible.

The followed approach aggregates activities by mapping them to the sub-process they belong to. Sub-processes are defined by some activities that work as separators and, in case the separation is not evident, process semantics. This mapping can be done by manually correlating each activity to its corresponding sub-process. Afterwards, an activity name substitution following the mapping will have a high-level event log as a result. The activities of this new event log will be the sub-processes comprising the activities of the old low-level event log. By reducing the number of activities, a process model that is simpler to analyze is obtained.

A manual strategy was used to define the sub-processes. It was done based on the knowledge of domain experts. The experts helped us to aggregate the activities, generating a sub-processes event log, which was then analyzed using process mining. A knowledge-based generation was preferred instead of using automatic techniques, such as trace clustering [24], because they do not perform well when there is excessive parallelism, since they are based on finding patterns of sequences of activities that repeat themselves in different episodes.

### 3.3. Phase 3: Filtering

After aggregating activities into sub-processes and generating the high-level event log, the next step is filtering the event log to reduce noise. There are several activities that are either not relevant to the entire process (but are still registered because they contain descriptive information) or are added to the historical record only for information storage purposes, thus not giving additional process input. These activities are identified using expert knowledge to directly discard them and by filtering out activities that follow highly uncommon paths or have a low frequency. As it is shown in Figure 1, Phase 3 is revisited later to generate specific process event logs depending on the goal of the process analysis. The filtered high-level event log retains all attributes coming from the low-level source event log. Using the attributes enumerated at the event log schema it is possible to filter the event log to generate sub-event logs that lead to specific process models that can answer questions related to process behavior depending on Triage color, Diagnosis, length of stay (duration), frequency of a path, among others. Using expert insight and event log filtering, it is possible to identify more general sub-processes (e.g., Discharge sub-process) that allow different levels of description of an ER episode. The main goal is to identify the episode paths that take a longer time to complete, thus identifying process bottlenecks that are not part of the knowledge of the episode that the process owners already have.

Disco was the tool of choice for filtering the event log due to its multiple filtering features and its ease of use. By using Disco filters, it is possible to filter the event log by activities allowing analysis of the event log for a specific stage of the episode, i.e., creating sub-logs to analyze the process per sub-process. Moreover, Disco allows filtering by attributes, which is useful to generate a derived event log for some specific type of episodes (e.g., event log with Red Triage color episodes only). Additionally, filtering the event log by episode duration is a straightforward task using Disco, resulting in sub-logs with different episode lengths of stay. Keeping this in mind, the event log filters allow generating process models from the first activity in the attention box until the patient is discharged, considering different Triage colors (different episode severity), or different lengths of stay, among others.

### 3.4. Phase 4: Discovery

Using the filtered event logs, process models are generated in Disco (or any other process mining tool, e.g., ProM (http://www.promtools.org) to be able to look in depth and analyze the behavior of the process. Depending on the filters applied in Phase 3, different models are obtained. In this phase, numerical information can be collected for different process variants. The goal is to collect detailed information of the process variants to elaborate a comprehensive analysis during the next phase. To build process models from filtered event logs, process mining discovery algorithms go through the event log and maps it onto a process model such that the model is “representative” of the behavior seen in the event log [5]. Given that the ER process has a spaghetti nature, the most suitable discovery algorithms are fuzzy and heuristic mining algorithms. The models created by Disco, called simply Process Maps, are based on the Fuzzy mining approach, which provides an extensible set of parameters to determine which nodes (activities) and arcs (dependencies between two activities) need to be included. Fuzzy models can be useful because they are able to abstract from details and aggregate process behavior that is not of interest depending on given parameters [25]. Fuzzy models and Disco’s process maps are not the best models to interpret, because they do not provide explicit elements to describe control flow, such as alternative or parallel paths. Another method to obtain process models is through Heuristic mining techniques, that consider frequencies of events and sequences into account when constructing a process model, resulting in an heuristic model that is not a perfect representation of the process behavior, but it is practical since it omits infrequent paths and infrequent activities [26]. Different techniques and tools provide different type of models, each with its own representational bias (e.g., Petri nets, Heuristics nets, Process Trees, among others). Given the interdisciplinary nature of this case study, we opted for Disco’s process maps, a process modeling notation that not necessarily has a formal semantic, but can be easily interpreted by medical stakeholders. As a result of the case study, we do not pretend to suggest that the process maps are the best representation, but in our case and based on previous studies [8], this is one of the most frequently used when applying process mining in healthcare.

### 3.5. Phase 5: Analysis

The analysis phase is required to study the resulting data and models. Being this a method to explore performance analysis, the analysis will provide information to the user regarding how the executed activities, the order in which they are performed and the number of times they are executed affect the process performance, e.g., why some episodes take longer than expected.

Inspecting the process model makes it easier to identify sub-processes by finding exit activities (i.e., activities that are frequently the end of most paths of a section of a process). Before analyzing, it is necessary to get the correct model by filtering the event log according to what is going to be analyzed and then discovering a new process model, which means that there is a need to go back to Phase 3 and 4 to filter the event log and generate the corresponding model. Figure 1 shows this procedure with an arrow starting at Phase 5 and going back to Phase 3, representing the possibility to iterate through Phases 3, 4, and 5 to perform a comprehensive analysis of the episode behavior.

### 3.6. Phase 6: Results and Evaluation

This last step considers the delivery of process models for different stages of the process, summarizing the data extracted from process models and comparison charts for process model duration, and performing validation and evaluation of these results and conclusions through expert opinion.

## 4. Case Study

Using the method described in the previous section, a case study was conducted at the Clinical Hospital of Red de Salud UC CHRISTUS, using historical data collected at its emergency room during July 2014. The hospital is a 500-bed academic medical center located in Santiago, Chile.

**Figure 3:**
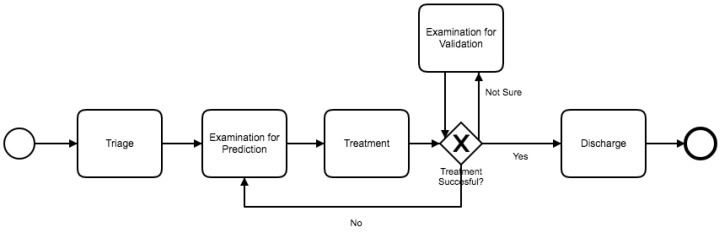
Emergency Room (ER) process: high-level BPMN model with the expected high-level process flow.

Figure 3 shows a BPMN diagram (See http://www.bpmn.org) of the expected high-level flow of the process according to experts for the ER in which the case study was conducted. A Triage sub-process and a Treatment sub-process were identified. Once a Triage sub-process was found, a deeper analysis using the triage color attribute was performed. After the Triage sub-process finishes, the rest of the process corresponds to the Treatment and Discharge sub-processes. Identifying the Discharge sub-process is simple since discharging a patient is a straightforward action. The Treatment sub-process involves most of the activities for each case. The conversion from low-level to high-level event log will collapse most activities depending on the nature of the tasks performed by the resources of the ER. There are examination activities that figure out what is the problem of the patient based on physical examination and tests, and to confirm if the patient is ready to be discharged. There are also treatment activities that are performed to lead the patient to a stable condition. These collapsed activities represent the Examination for Prediction, Treatment and Examination for Validation sub-processes. Whenever the treatment is not successful, another instance of Examination for Prediction starts, followed by Treatment. In some cases, Examination for Validation is performed by medical order. Analyzing the duration of these sub-processes provides insight about the behavior of the process and how performance of these sub-processes affect the whole process. Analyzing the Treatment models through filtering by duration defines differences between short stays and long stays. Finally, considering referrals to specialized physicians provides information about duration of the process depending on the complexity of the diagnosis. A detailed description of the executed tasks performed at each Phase of the study is provided next.

## 5. Results

### 5.1. Phase 1: Extraction and Transformation

The data used to construct the event log is historical data collected by the research team from the Hospital Information Systems Alert ADW Phase I, which is the system used to store the data from the different activities in the ER episodes. In the system, an ER episode is created for each patient. The ethical committee of the hospital authorized the use of the data for research purposes. Data were extracted as a single Excel file, where each row represents a single documented activity in a given episode. Historical data were built as an enumeration of 309,796 activities registered into the system, composing 7160 different episodes. There are 64 different activity types registered. Each register includes its timestamp, its resource, and the episode ID. In addition to these attributes, the analysis considers diagnosis, type of resource (nurse, physician, technician, auxiliary nurse) and triage color. It is worth noting that the activity timestamps are registered at the hour level. However, timestamps of the first and the last activity are registered considering minutes and seconds. Statistical results are analyzed having this level of granularity in consideration. By selecting the corresponding attributes using Disco’s import tool, an event log is built based on this historical data.

### 5.2. Phase 2: Activity Aggregation

Disco was used to filter the event log according to the opinion of a medical expert, who explained that the activities could be classified in 3 sub-processes: Triage, Examination–Treatment, and Discharge activities. These groups are initially considered as sub-processes of the ER episode. Triage activities are all the activities performed before “First Triage” activity. Discharge activities are the ones at the end of the process, include “discharge” in their name, and were also identified by the expert. Examination–Treatment activities are diverse and there are many of them with different possible paths. A complex process model is expected for the Examination–Treatment sub-process. Since there are several different Examination and Treatment activities, there is a need to group them into higher-level activities that represent a wider concept of what has been done, so as to create a more accurate model.

Aggregation of activities into sub-processes is based on expert opinion and observation of the process. Several activities were not considered to be relevant by the domain expert, so they were not mapped to any sub-process. Each of the relevant activities is mapped to a collapsed activity, i.e., a sub-process, by enriching the event log with this information. Table 2 shows the relationships created to achieve this representation for all sub-processes. Activities that are not included in this table are disregarded because they are not providing relevant information to the process model, according to the expert.

Concerning the Examination sub-process, activities could be of two types: “for prediction” or “for validation”. Examination for prediction is the first examination, performed by a physician, that provides enough information to figure out what problem the patient has. Examination for validation is an activity, also performed by a physician, that is done with the goal to check that everything is fine with the patient and that there is no need to remain in the ER. To make a difference between both types it is necessary to check the whole current episode. If current examination is the last one in the specific episode and not the first one, it is considered an examination for validation, otherwise it is an examination for prediction. Identifying types of examination was performed using a script implemented for this purpose. The described procedure is formalized in Algorithm 1.

 **Algorithm 1:** Identify examination type.  
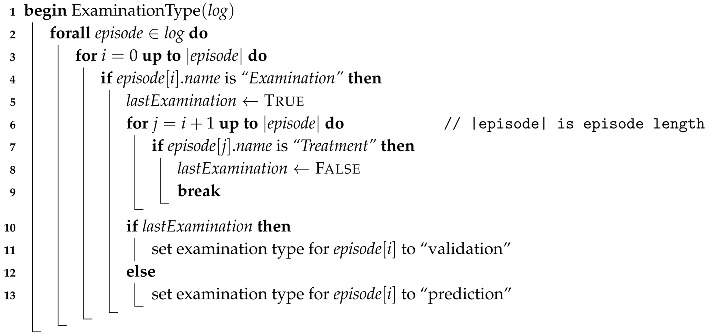


### 5.3. Phase 3: Filtering

At the first filtering phase, events that were not mapped to any sub-processes are removed using Disco filtering capabilities. This filter removes noise and paths that are not required to analyze due to their low frequency. For the following iterations of the filtering phase, additional filters are applied to generate specific process models for the case study. In our case, the filtering was done by starting and ending points, by performance results, and by episode attributes. Filtering by starting and ending points allows getting a process model that does not consider terminated episodes that do not end with the usual patient discharge. Filtering by performance results allows getting process models that represent different behaviors depending on how long does it take to finish the episode. This filter is useful to find differences between slow and fast cases. Filtering by attributes, other than by name (e.g., Triage color), makes possible to identify differences between episodes that have different characteristics, and how these differences affect the episode.

### 5.4. Phase 4: Discovery

**Figure 4:**
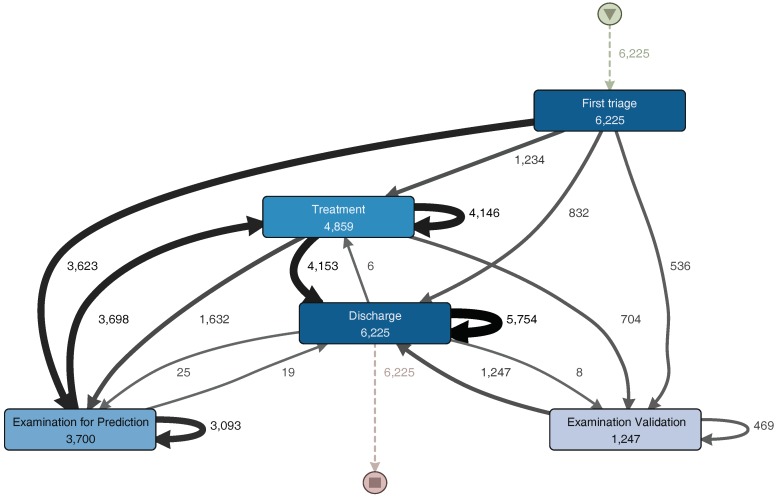
Process model for the enriched event log. A darker node color represents a higher number of episodes that went through a sub-process. The thickness of the arrows represents the number of episodes that had paths between two sub-processes. Numbers represent the number of episodes in which each sub-process or path occurred. (Disco parameters: activities: 100%; paths: 0%; show: episode frequency).

Once a filter is applied, Disco generates a process model that represents the portion of the event log resulting from the event log aggregation and filtering [27]. Disco also provides performance analysis, but this did not provide significant results for the analysis. From the sub-process event log, we obtain the process model illustrated in Figure 4. The generated model is similar to the one previously shown in Figure 3. The main trajectory followed by the episodes is: First triage–Examination for Prediction–Treatment–Discharge. Notice that some sub-processes present a self-loop arch, indicating that those sub-processes are repeated in some episodes. The main difference between these two models is that Figure 4 includes infrequent paths (e.g., episodes where the patient is taken back from Discharge to Treatment). A path is considered less frequent when the number of episodes that followed that path is less than a certain threshold. In Disco, that threshold can be specified using a specific parameter that goes from 0% (only the most frequent paths are shown in the diagram) to 100% (all paths are shown in the diagram).

The analysis of the Triage sub-process was done separately since it is the starting point of the ER process, and it does not present significant increments in time for the episodes. Meanwhile the examination, treatment and discharge sub-processes were analyzed together to identify repetitions and possible relationships between them, given that these three sub-processes happen at the attention box and are performed by the same resources.

**Figure 5:**
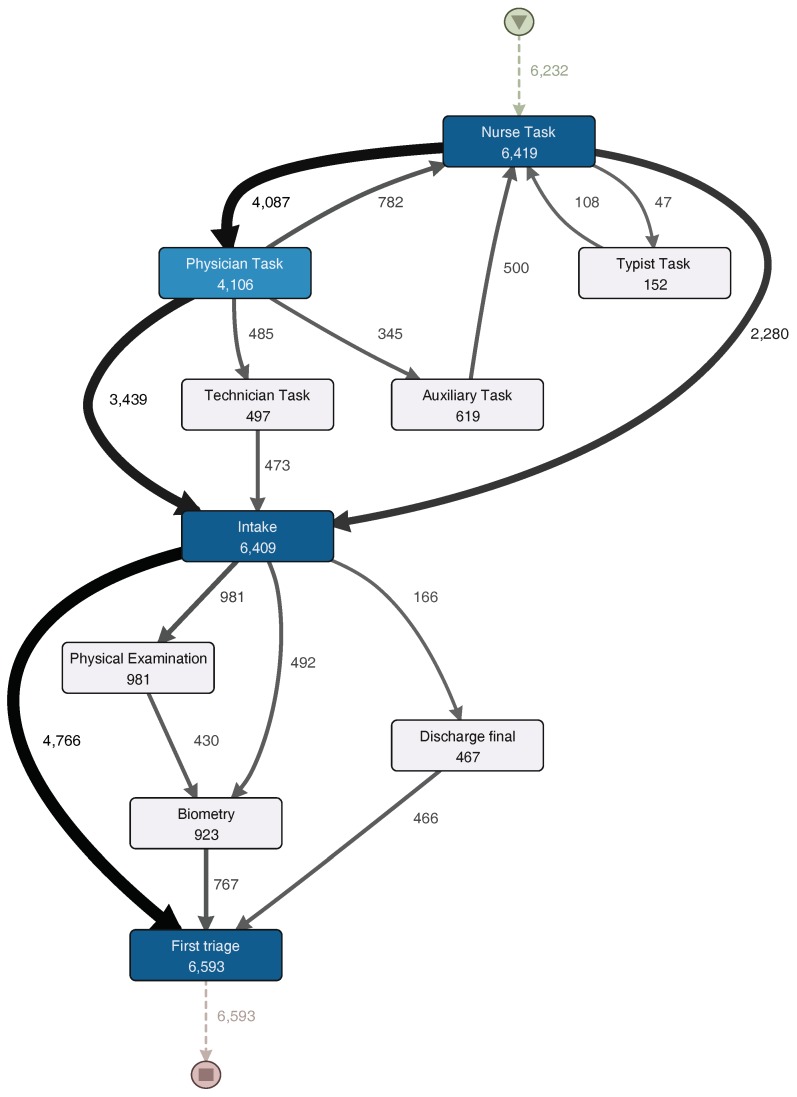
Triage Sub-Process Model. A darker node color represents a higher number of episodes in which each activity was performed. The thickness of the arrows represents the number of episodes that had paths between two activities. Numbers represent the frequency of each activity or path. Infrequent paths are filter out to reduce the complexity of the model. (Disco parameters: activities: 100%; paths: 0%; show: episode frequency).

**Figure 6:**
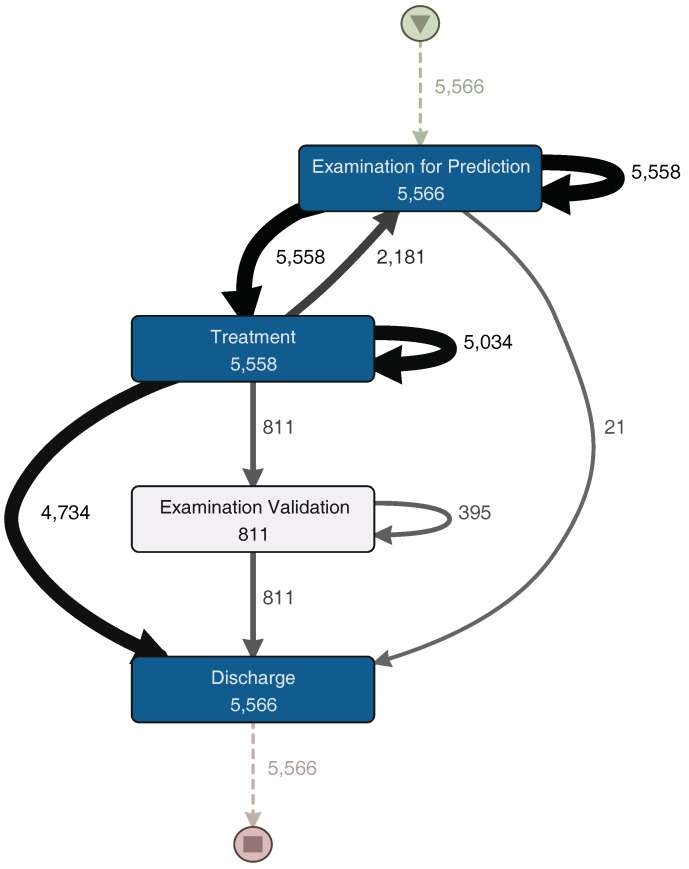
Process model for the Examination–Treatment–Discharge (ETD) sub-process. A darker node color represents a higher number of episodes in which each activity was performed. The thickness of the arrows represents the number of episodes that had paths between two activities. Numbers represent the frequency of each activity or path. (Disco parameters: activities: 100%; paths: 0%; show: episode frequency).

Figure 5 shows a process model with the activities executed during the Triage sub-process, while Figure 6 shows a process model with the activities executed during the examination, treatment and discharge sub-processes. They will both be analyzed in the description of the following phase.

### 5.5. Phase 5: Analysis

Next, the analysis of the different sub-processes will be described.

#### 5.5.1. Triage Sub-Process

The initial sub-process in each ER episode seeks to determine the severity of each episode according to the patient’s conditions. Usually, it consists of categorizing each patient in one of the Manchester triage categories (red, orange, yellow, green and blue) [28]. Several activities were selected by the ER specialist from the event log to see the process model followed in the analyzed episodes.

The Triage sub-process normally starts with a “Nurse task” activity. After that, the process continues to a “Physician task” and then to an “Intake task” activity. In one third of the episodes, a physician does not take part in the triage sub-process and the patient goes directly to “Intake task”. About 10% of the episodes that go through the physician are then taken by a technician (“Technician task”), and then an intake note is generated. After the generation of the intake note, a physical examination could occur or vital signs could be registered (these activities could be skipped). Finally, the first triage is done, and the triage sub-process is finished.

For a more detailed analysis of the process, the event log was split in two groups: one half with the fastest episodes and the other half with the slowest episodes. However, no relevant differences in activities presence or their sequence were discovered.

#### 5.5.2. Examination, Treatment, and Discharge Sub-Process

The Examination, Treatment, and Discharge sub-process (ETD) includes three main subsets of activities. The first subset contains the activities related to the examination of the patient. The second subset is the treatment, including treatment activities, medications and exams. Finally, discharge activities, including paperwork and the actual patient discharge, are grouped into a third subset. Figure 6 shows a process model with the activities executed during the ETD sub-process. The Treatment sub-process begins with “Examination for Prediction”. This activity includes all nurse and physician activities that occur before any actual treatment is performed. After this examination, a consultation to a specialist could happen. Then, the process continues with the actual treatment. After the treatment is finished, the patient is discharged. Also, a loop between examination for prediction and treatment is identified. Subsequently, after the patient is treated, a physician could request a final examination to validate whether the patient could be discharged or not. It is worth noting that the difference between short and long episodes depends almost exclusively of this sub-process. Long episodes of the complete process are the ones that include several of these “examination for prediction–treatment” repetitions. Finally, patient discharge is straightforward. After aggregating the activities into one general discharge activity, the sub-process was collapsed into only one activity. The process structure is simple and sequential. Each activity is directly followed by the previous one with no alternative paths.

**Figure 7:**
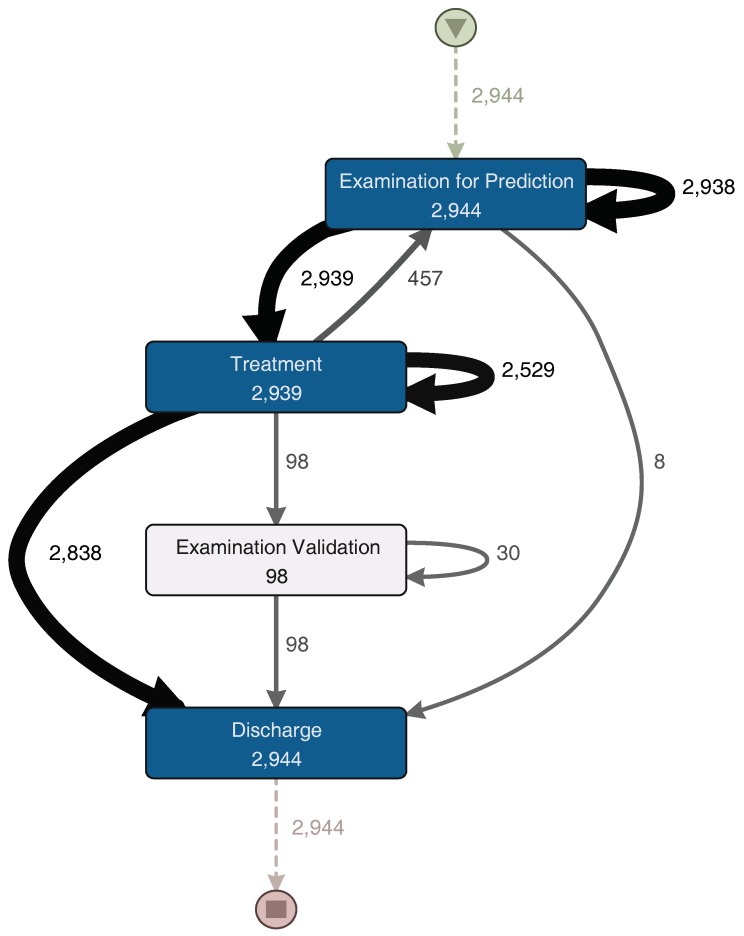
ETD—fast episodes. A darker node color represents a higher number of episodes in which each activity was performed. The thickness of the arrows represents the number of episodes that had paths between two activities. Numbers represent the frequency of each activity or path. (Disco parameters: activities: 100%; paths: 0%; show: episode frequency).

**Figure 8:**
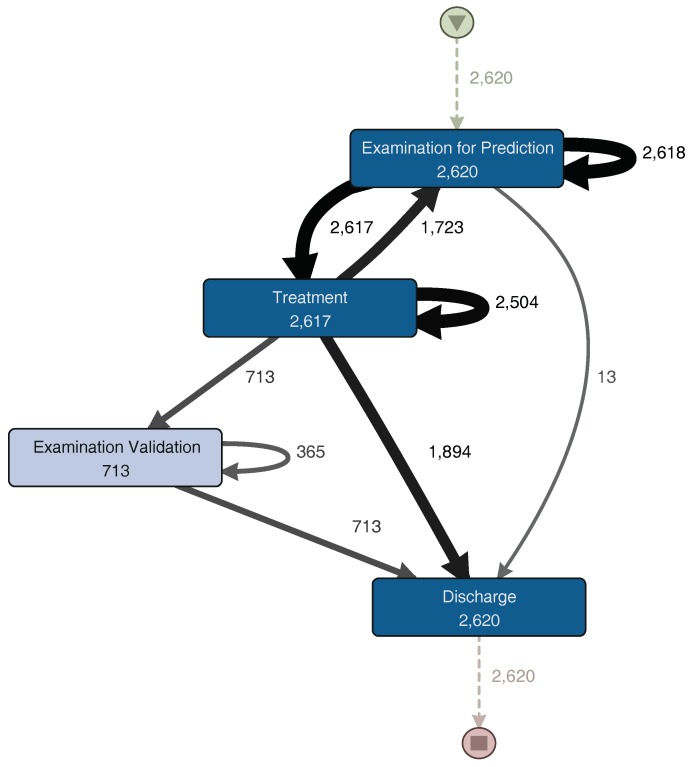
ETD–slow episodes. A darker node color represents a higher number of episodes in which each activity was performed. The thickness of the arrows represents the number of episodes that had paths between two activities. Numbers represent the frequency of each activity or path. (Disco parameters: activities: 100%; paths: 0%; show: episode frequency).

For a more detailed analysis of the process, the event log was split into two groups. The first group corresponds to the half with the fastest episodes. In Figure 7, the process model for these episodes is shown. The other group includes the half with the slowest episodes; its process model can be seen in Figure 8. The “Examination Validation” sub-process is more common in the slower episodes, since it is present in 27.2% (713 of 2620 episodes) of them. Therefore, it is important to study and identify why it occurs, evaluate its contribution, or the time it takes to perform it. In addition, a loop between the “Examination for Prediction” and the “Treatment” sub-processes was identified on both process models (Figure 7 and Figure 8). This loop could occur several times in one episode. Every time this loop occurs, the duration of the episode increases. The loop is present in 65.8% of the slowest episodes (1723 of 2620 episodes), while it is only present in 15.5% (457 of 2944 episodes) of the fastest episodes. As part of this case study, we will now focus on understanding what characteristics have in common the episodes that include this loop.

#### 5.5.3. Analysis by Triage Color

A relevant case attribute for this process is Triage color since it informs resources about the severity of the patient case [28,29]. A characterization of the process based on this attribute might help to understand how the different episodes are handled. Table 3 shows a detailed description of the characteristics of the Examination for prediction–Treatment loop, described in the previous analysis. In general, higher priority episodes have a higher average of repetitions, being a repetition an execution of the Examination for prediction–Treatment loop. Orange and red episodes involve more complex diagnoses and treatments which explains that in average these episodes have more than two repetitions of the Examination for prediction–Treatment loop. Green episodes, in average, have half the number of repetitions (1.27 average repetitions) of orange episodes (2.34 average repetitions).

**Figure 9:**
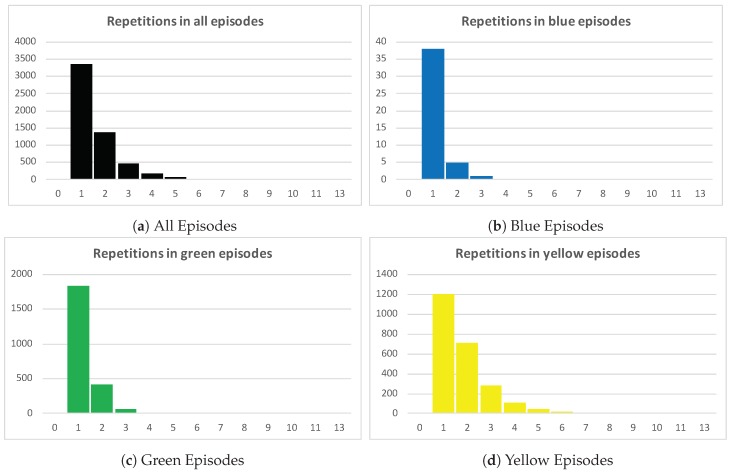

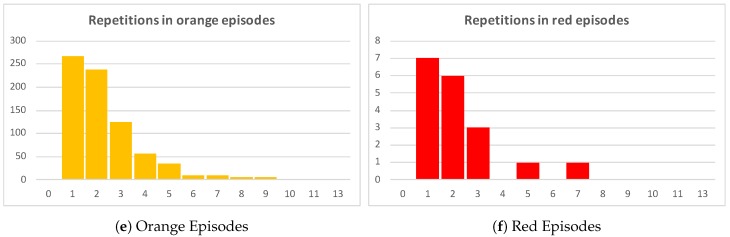
Repetition of Examination for prediction–Treatment loop by triage color.

**Figure 10:**
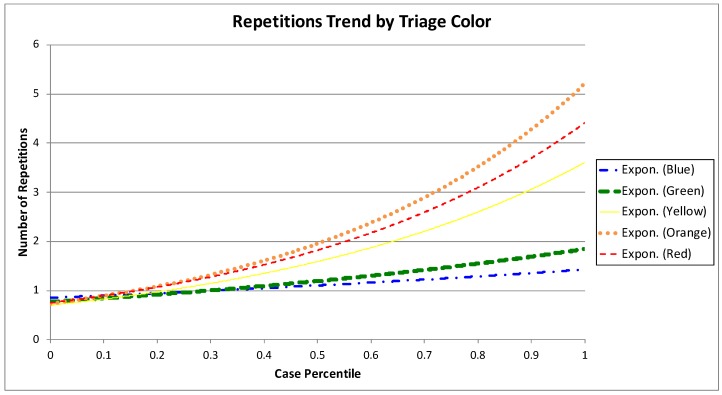
Number of repetitions trend of the Examination for prediction–Treatment loop by triage color.

Figure 9 show these results. Figure 9a includes all episodes of the event log and the number of repetitions of each episode plotted in black. By simply inspecting the chart, it is clear that more than one third of total episodes have at least two repetitions of the Examination–Treatment loop. The other Figure 9b–f show episodes grouped by triage color. Figure 9c groups green episodes, where just about a quarter of the episodes have two or more repetitions. Figure 9d,e make clear that the portion of episodes with two or more repetitions increases as the severity of the episode is higher. The number of repetitions trend by triage color is shown in Figure 10. The trends of higher severity episodes are more steep, which enforces previously shown results. For example, the trend for green episodes moves between one and two repetitions, while the trend for orange episodes is between one and six repetitions.

#### 5.5.4. Analysis by Discharge Disposition

An additional exploratory analysis was also performed using the discharge disposition attribute for all the episodes. The objective was to determine whether the presence of the Examination for prediction–Treatment loop was similar for the different types of episodes, according to their discharge disposition.

Table 4 shows there are six different discharge dispositions for the analyzed episodes. We will focus the analysis on the four more relevant disposition (Regular discharge, Discharge with ambulatory follow-ups, Abandonment, and Hospitalization), that include 6389 (98%) of episodes and 3517 (97.3%) of Examination for prediction–Treatment repetitions. The episodes with highest percentage of Examination for prediction–Treatment repetitions are the episodes that lead to Hospitalization as the discharge disposition, including almost 44% of the repetitions. Regular discharge and Discharge with ambulatory follow-ups episodes include around 26% of the repetitions each.

Episodes with Hospitalization as discharge disposition correspond to only 11.43% of the total amount of episodes, but include 44.25% of the Examination for prediction–Treatment repetitions. This is relevant and must be studied with more details, so as to identify what characteristics of these episodes make them have more repetitions. As an initial analysis, a description per diagnosis was performed for these episodes. The top six diagnoses for the episodes that present the Examination for prediction–Treatment loop within the hospitalized patients are shown in Table 5, with Pneumonia and Sepsis being the most relevant ones.

### 5.6. Phase 6: Results and Evaluation

By analyzing the generated process models with ER experts, there are two situations where the ER process slows its pace, increasing waiting times. The first situation is at the examination–treatment sub-process. Depending on how many times the patient is examined to detect what their problem is (the process extends per each examination–treatment loop), the total duration increases. The other situation where the process takes longer than expected is when examination for validation takes place, extending the duration by one up to two hours (on average).

There is a relationship between triage color and the number of examination–treatment repetitions. As the severity of the episode increases, the number of repetitions increases as well, showing that Blue and Green episodes (low severity) have a below average number of repetitions while Yellow, Orange, and Red episodes (high severity) have a number of repetitions above the average. Triage color does not affect any other sub-process of the ER process.

Regarding discharge disposition, Hospitalization is the major discharge disposition for episodes with higher number of the examination–treatment loop, including episodes with diagnoses such as Pneumonia, Septicemia, Severe respiratory insufficiency, among others.

## 6. Discussion

The discussion will be carried out in two perspectives, first from the Emergency Room perspective, then from the Process Mining perspective.

Emergency Room perspective. Performance directly impacts episode duration and improves resource usage in the ER. By identifying longer episodes and characterizing them through process mining techniques, critical paths can be discovered and considered when improving ER services. Although not an explicit component of this study, we validated the results with expert clinicians. Visual representations of the real-world processes observed in the ER provided relevant information that could, in the future, generate changes in the disposition of patients. For example, under high-demand situations, patients undergoing a second or third iteration of the Examination for prediction-Treatment loop might be transferred to a separate unit so as to not block patient flow. This idea may need to be empirically tested and validated in the future. In this article, several sub-processes and their relationships were identified. From the ER point of view, this helps to see which activities implicate an increment on the episode duration. The increment of episode duration directly increases the waiting times for all the episodes, and increments the ER overcrowding, which has become one of the most relevant issues nowadays [30].

Four sub-processes were identified, but two main sub-processes are critical. First, the Examination sub-process, which includes activities where the resources try to determine the diagnostic of the patients to take actions to bring care to the patients. Secondly, the Treatment sub-process where any exam, procedure, or medication is provided. In this case study, through process mining, a loop between the two sub-processes was identified. Every time it happens, time is added to the episode duration. It was also analyzed according to the different triage colors, discharge dispositions, and diagnoses, giving a more detailed description on when this loop happens more often, so as to pay more attention in future episodes. Being able to identify episodes where this loop between the two sub-processes might happen can help physicians, nurses, technicians, and ER process experts to improve their labor significantly.

Process Mining perspective. From the process mining perspective, the proposed case study is significant to the field. Two levels of analysis were executed. First, a low level detailed analysis was performed with the domain expert to identify the executed activities and the sub-processes to which they belong. Secondly, a high level analysis was carried out to see the relationships between the different sub-processes so as to identify which are the causes of slowest episodes.

The lessons learned from a process mining perspective are:

- As it is well-known, in a spaghetti-like process as the one performed in the ER episodes is not feasible to obtain a single meaningful model that describes all the process behavior. In this case study, we show it is relevant to analyze the process at different levels of abstraction Two levels of analysis were executed. A low level detailed analysis was performed with the domain expert to identify the executed activities and the sub-processes to which they belong. A high level analysis was carried out to observe the relationships between the different sub-processes, so as to identify which are the causes of slowest episodes.
- Even though trace clustering and other techniques can be applied to group activities, in this case it was more effective to group activities following the recommendations of domain experts. It may be more laborious, but the results are more meaningful.
- The granularity of the timestamp must be treated carefully. Obviously, the more accurate the timestamp is, the greater the analysis that can be obtained. It is also important that all timestamps have the same level of accuracy. In this case, we had timestamps at different levels of granularity, so we could not use them to apply statistical performance analyzes. That is why we used the number of repetitions as a proxy of process delay.
- Grouping traces can help to improve performance analysis. In this case, we opted for a grouping based on the knowledge of the domain expert, which allowed us to better understand how the case severity affects the duration of the process.
- The use of an iterative approach to apply process mining in health care provides the opportunity to improve the results and take into account the feedback of domain experts.

## 7. Conclusions and Future Work

By analyzing the ER process, it was discovered that the loop between the Examination and Treatment sub-processes increments the duration of ER episodes. This will help with the identification of the episodes where this loop happens (mainly by triage color, discharge disposition, and some diagnoses), to make any necessary improvements. Identifying this loop as the main cause of the increment in the episode duration is significant to help reduce its occurrence, and in turn, lower the episode duration, free boxes in the ER, provide faster attention to more patients, shorten waiting times, and finally reduce the overcrowding in the ER. Further work will include applying additional process mining algorithms and more statistical techniques, in order to analyze an extended dataset of ER episodes, achieving a more in depth quantitative analysis. Furthermore, a more in depth research should be carried out on the relevance of the evidence provided by these models for the ER.

## Figures and Tables

**Table 1:** Event Log Schema. These are the attributes required by the method to build an Emergency Room (ER) event log.

Attribute
Episode ID
Activity Name
Timestamp
Resource
Resource Type
Diagnosis
Triage Color
Patient Destination

**Table 2:** Activity Aggregation mapping. Original Activity column shows the activity names extracted from the Hospital Information System (HIS). The sub-process column shows high-level activity names representing a sub-process.

Original Activity	Sub-Process
Nurse task	Examination
Physician task	Examination
Prescribe Medication	Treatment
Perform Procedures	Treatment
Physical Examination	Treatment
Give Medication	Treatment
Prescribed Procedures	Treatment
Prescribed Medication (internal)	Treatment
Required Laboratory Tests	Treatment
Required Imagenology Tests	Treatment
Biometry	Treatment
Other Required Tests	Treatment
Cancelled External Medication Prescription	Treatment
Final Discharge	Discharge
Clinic Discharge	Discharge
Last Discharge	Discharge

**Table 3:** Examination for prediction–Treatment loop characteristics by triage color.

	All	Blue	Green	Yellow	Orange	Red
Episode frequency	5538	44	2347	2374	755	18
Relative frequency	100%	0.56%	32.58%	47.1%	19.3%	0.44%
Total repetitions	9184	51	2292	4328	1773	40
Max repetitions	13	3	9	10	13	7
Average repetitions	1.66	1.16	1.27	1.82	2.34	2.22
2 or more repetitions	39%	13%	21%	29%	64%	61%
3 or more repetitions	14%	2%	4%	19%	32%	27%

**Table 4:** Examination for prediction–Treatment loop occurrences by discharge disposition.

Discharge Disposition	# Episodes	%	# Repetitions	%
Regular discharge	2705	41.55%	953	26.37%
Discharge with ambulatory follow-ups	2050	31.49%	965	26.70%
Abandonment	890	13.67%	0	0%
Hospitalization	744	11.43%	1599	44.24%
Discharge with ER follow-ups	85	1.31%	69	1.91%
Others	37	0.57%	28	0.77%

**Table 5:** Top diagnoses of patients that present the Examination for prediction–Treatment loop and have Hospitalization as discharge destination.

Diagnosis	# Episodes	# Repetitions
Pneumonia	37	109
Sepsis	23	81
Severe respiratory insufficiency	17	62
Acute appendicitis	27	50
Acute bronchiolitis	16	50
Unspecified fever	15	44
